# Guideline Adherence of Perioperative Antibiotics and Surgical Site Infections in Noncardiac Surgery

**DOI:** 10.1001/jamanetworkopen.2025.59349

**Published:** 2026-02-18

**Authors:** Amit Bardia, Hung-Mo Lin, Xiwen Zhao, George Michel, Mabel Wai, Clark Fisher, Kevin M. Shuster, Douglas A. Colquhoun, Michael R. Mathis, Michael McGee, Sachin Kheterpal, Jill Mhyre, Robert B. Schonberger

**Affiliations:** 1 Department of Anesthesia, Critical Care and Pain Medicine, Massachusetts General Hospital, Harvard Medical School, Boston; 2 Department of Anesthesiology, Yale School of Medicine, New Haven, Connecticut; 3 Yale Center for Analytical Sciences, Yale School of Public Health, New Haven, Connecticut; 4 Department of Pharmacy, Yale New Haven Hospital, New Haven, Connecticut; 5 Department of Surgery, Yale School of Medicine, New Haven, Connecticut; 6 Department of Anesthesiology, University of Michigan, Ann Arbor; 7 Department of Surgery, University of Michigan, Ann Arbor; 8 Department of Anesthesiology, University of Arkansas for Medical Sciences, Little Rock

## Abstract

**Importance:**

Despite nearly universal adherence to the Surgical Care Improvement Project (SCIP), surgical site infections (SSIs) persist. Compared with SCIP, which largely focuses on antibiotic timing, the Infectious Diseases Society of America (IDSA) guidelines provide a more comprehensive framework of antibiotic metrics, including procedure-specific antibiotic selection, weight-adjusted dosing, timing of the first dose, and appropriate redosing.

**Objective:**

To assess whether nonadherence to each antibiotic administration metric of IDSA guidelines is associated with SSIs.

**Design, Setting, and Participants:**

In this nationwide, multicenter, cross-sectional study, patients aged 18 years or older who underwent noncardiac surgeries involving a skin incision between January 1, 2014, and August 31, 2022, were included from merged data of the Multicenter Perioperative Outcomes Group, National Surgical Quality Improvement Program, and Michigan Surgical Quality Collaborative registries. Analyses were conducted between July 2, 2024, and April 24, 2025.

**Exposure:**

Nonadherence to IDSA-defined antibiotic metrics.

**Main Outcomes and Measures:**

The primary end point was SSI, defined as any superficial, deep tissue, or organ-space infection as recorded in the National Surgical Quality Improvement Program and Michigan Surgical Quality Collaborative registries. The association of nonadherence to IDSA guidelines (both overall and individually) was examined using hierarchical generalized linear mixed models.

**Results:**

Of 134 413 eligible surgical cases, a total of 119 236 patients (mean [SD] age, 56.2 [15.9] years; 58.1% women) from 37 institutions met the inclusion criteria, among whom 6796 (5.7%) had incomplete covariate data. Failure to adhere to any IDSA metric was common in 26.1% of cases, with individual nonadherence rates as follows: 13.3% for antibiotic choice, 9.0% for weight-adjusted dosing, 3.0% for timing relative to incision, and 4.8% for correct intraoperative redosing interval. Overall, SSIs occurred in 4.4% of cases. After adjusted analysis, guideline-nonadherent antibiotic administration was significantly associated with SSIs (relative risk [RR], 1.34 [95% CI, 1.26-1.43]). Nonadherence to antibiotic choice (RR, 1.43 [95% CI, 1.33-1.53]) and failure to appropriately redose intraoperatively (RR, 1.12 [95% CI, 1.02-1.24]) were significantly associated with SSIs.

**Conclusions and Relevance:**

This cross-sectional study found that IDSA guideline nonadherence, including incorrect antibiotic choice and missed intraoperative redosing, was common and associated with increased SSI risk, despite high adherence to SCIP timing metrics. Improving adherence to IDSA-recommended antibiotic selection and redosing may meaningfully reduce SSIs.

## Key Points

**Question:**

What is the association of adherence to Infectious Diseases Society of America (IDSA) perioperative antibiotic prophylaxis guidelines, covering antibiotic choice, dosing, timing, and intraoperative redosing, with surgical site infections (SSIs) after noncardiac surgery?

**Findings:**

In this cross-sectional study of 119 236 patients who underwent noncardiac surgery across 37 US institutions, nonadherence to at least 1 IDSA antibiotic metric occurred in 26.1% and was associated with higher SSI incidence (4.4% overall). Nonadherence to antibiotic choice and failure to redose intraoperatively were each independently associated with increased SSI risk.

**Meaning:**

These findings suggest that improving adherence to IDSA antibiotic prophylaxis guidelines, particularly for antibiotic selection and redosing, may reduce SSIs and improve surgical outcomes.

## Teaser

This cross-sectional study evaluates how many institutions adhere to Infectious Diseases Society of America guidelines for perioperative antibiotics and how often surgical site infections (SSIs) occur.

## Introduction

Surgical site infections (SSIs) are the leading cause of health care–associated infections in surgical patients, affecting more than 160 000 patients annually.^1,2^ Beyond outcomes associated with quality of life and morbidity, the financial burden incurred is estimated to be $3.5 billion each year in the US.^3^ Despite numerous interventions to reduce SSIs, they continue to be a considerable health care issue.^4^

It is estimated that nearly one-half of SSIs are preventable, and one potential way to prevent them is by increasing adherence to already-established best practices for antimicrobial prophylaxis.^4,5^ Numerous studies have explored the role of perioperative antibiotic prophylaxis in preventing SSIs, yet substantial knowledge and practice gaps persist.^6,7,8^ In response to stagnant SSI rates, the Infectious Diseases Society of America (IDSA) led the development of comprehensive perioperative antibiotic prophylaxis guidelines in 2013, which were endorsed by multiple stakeholders and are more detailed than the widely recognized Surgical Care Improvement Project (SCIP) guidelines.^9^ However, implementation of the IDSA guidelines has been poor due to limited publicity and a lack of robust data supporting their effectiveness.^10^

A multicenter observational study found that 35.9% of adult patients undergoing general, urologic, orthopedic, and gynecologic surgeries between 2014 and 2018 received antibiotics nonadherent to at least 1 aspect of the IDSA guidelines regarding type, dose, timing, and/or intraoperative redosing of guideline-concordant antibiotics.^11^ Small studies examining various components of these guidelines have found that overall nonadherence is associated with an increased risk of SSIs.^6,7,12,13^ However, further research is needed to clarify the overall impact of the different components of IDSA guideline adherence on SSIs using highly detailed nationwide data, as well as to identify factors contributing to nonadherence and the development of strategies to improve adherence.

Accordingly, this study aimed to evaluate the association between adherence to individual components of the IDSA perioperative antibiotic prophylaxis guidelines, including antibiotic selection, weight-based dosing, timing of administration, and intraoperative redosing, and the risk of SSI after noncardiac surgery. We hypothesized that nonadherence to 1 or more of these antibiotic administration metrics would be associated with a higher incidence of SSIs. To address this knowledge gap, we used a combination of detailed intraoperative medication selection, dosing, and administration timing data with high-fidelity SSI outcomes data to measure the possible association between each component of IDSA nonadherence and subsequent adjudicated SSIs.

## Methods

This cross-sectional study used data from the Multicenter Perioperative Outcomes Group (MPOG), American College of Surgeons National Surgical Quality Improvement Program (NSQIP), and Michigan Surgical Quality Collaborative (MSQC) databases. The MPOG Perioperative Clinical Research Committee approved the protocol, including an a priori plan of data access and analysis, as did the Yale Institutional Review Board with a waiver of the requirement of informed consent because of the use of routinely collected health data. The study followed the Reporting of Studies Conducted Using Observational Routinely Collected Health Data (RECORD) reporting guideline.^14^

The MPOG registry is one of the largest perioperative databases in the US, comprising more than 12 million anesthetic cases from more than 50 medical centers. It captures highly granular, time-stamped intraoperative data, including medication administration, physiologic parameters, ventilation details, and procedural timelines, from 4 hours before anesthesia start to 6 hours after anesthesia end. All cases requiring anesthetic care were included without sampling. Each contributing institution performed rigorous data cleaning, and a standardized, deidentified dataset was centrally uploaded to the MPOG registry. The NSQIP is a nationwide, nurse-adjudicated clinical registry with more than 700 participating hospitals and more than 11 million historical cases. Trained clinical abstractors collect standardized preoperative risk factors and 30-day postoperative outcomes, including superficial, deep, and organ-space SSIs, using validated definitions and strict auditing procedures. The NSQIP is considered the criterion standard for high-fidelity SSI outcome ascertainment. The MSQC is a statewide quality improvement consortium of 69 hospitals that uses the same standardized methods as NSQIP but with additional regional support, real-time data validation, and enhanced auditing. It similarly provides 30-day postoperative SSI follow-up data collected by trained abstractors. Together, these registries provide complementary strengths: MPOG offers detailed, high-resolution intraoperative antibiotic administration data, whereas NSQIP and MSQC supply rigorously adjudicated, complete 30-day SSI outcomes, allowing for robust linkage between perioperative antibiotic practices and postoperative infection events.

Participating centers linked their local NSQIP and/or MSQC data to local MPOG surgical case-level data, which were then uploaded monthly to the centralized MPOG server. Data from MSQC were integrated centrally based on matching case dates, patient age, and sex. Since the IDSA guidelines were proposed in February 2013, data from January 1, 2014, to August 31, 2022, were extracted from the MPOG database.

### Study Population

The study included patients aged 18 years or older who underwent noncardiac surgery involving a skin incision from January 1, 2014, through August 31, 2022, and had MPOG data available, as well as data on SSI status from either the NSQIP dataset or the MSQC dataset. Patients with the following characteristics were excluded: preoperative open wound with or without infection, missing intraoperative time or dose of antibiotic documentation, missing weight, missing surgical *Current Procedure Terminology* code, and an American Society of Anesthesiologists (ASA) score of 6. We further excluded cardiac surgeries, organ transplant surgeries, ophthalmic surgeries, and procedures without skin incision (eFigure 1 in Supplement 1). In this analysis, each observation represented a surgical case rather than a unique patient, as individuals could contribute more than 1 case provided that procedures occurred more than 30 days apart. To avoid misclassification of postoperative events, only the index case was included for patients undergoing multiple operations within any 30-day period. Consistent with NSQIP and MSQC methodology, all cases were followed up for 30 days postoperatively to ascertain SSI, with this follow-up interval standardized across all participating institutions.

### Exposure and End Points

The exposure of interest was any nonadherence to the IDSA-defined antibiotic metrics, both in isolation or in combination, with respect to antibiotic choice, weight-based dosing, timing with respect to incision, or appropriate redosing (definitions provided in the eMethods in Supplement 1). To ensure data fidelity, only cases with complete and validated time-stamped documentation of antibiotic administration were included. The MPOG medication timing fields undergo routine institutional and central quality checks, providing reasonable reliability of this variable.

For each *Current Procedure Terminology *grouping, we constructed procedure-specific lists of IDSA-recommended first-line and acceptable alternative agents. All perioperative antibiotics, including primary and secondary or adjunct agents, were mapped to these lists. Cases were classified as nonadherent only when none of the administered agents (alone or in combination) met the guideline-recommended regimen for that procedure, including procedures requiring multiagent prophylaxis.

The primary end point was SSI, which was defined as any superficial, deep tissue, or organ-space infection as recorded in the NSQIP or MSQC registries within 30 days postoperatively. Both NSQIP and MSQC use validated and cross-checked methodologies for collection of outcomes as described elsewhere.^15,16^

### Covariates

Covariates were extracted for each surgical episode. The following covariates were collected: demographic data (age, sex, self-identified race [Black, White, other (American Indian or Alaska Native, Asian, Native Hawaiian or Pacific Islander) or unknown] and Hispanic ethnicity); anthropometric measurements (weight, height); smoking status (current vs not current); ASA physical status score; van Walraven comorbidity index (calculated based on the presence of administrative codes for Elixhauser comorbidities^17,18,19^); cerebrovascular disease; diabetes; surgical characteristics (emergency vs nonemergency surgery, whether the surgery was performed during the evening or night [5:00 pm to 6:30 am]); year of surgery; duration of anesthesia; center (teaching vs community hospital based on medical school affiliation); and intraoperative variables, including hypotension (calculated as the time in minutes of mean arterial pressure <55 mm Hg as measured throughout the intraoperative period) and whether the patient received blood product transfusions. Race and ethnicity were chosen a priori to address confounding related to these variables.

### Statistical Analysis

Descriptive statistics for continuous variables were selected based on their distribution; variables that were approximately normally distributed are presented as mean (SD), whereas skewed variables are presented as median (IQR). For categorical variables, descriptive statistics included counts and percentages. Histograms and box plots were constructed to evaluate the distributions of continuous variables and identify potential outliers. Unadjusted group comparisons were performed using χ^2^ tests or Fisher exact tests for categorical variables and *t* tests or Kruskal-Wallis tests for continuous variables based on distributions.

A 2-sided *P* < .05 was considered statistically significant, unless otherwise noted. Appropriate effect sizes, such as relative risk (RR), and their corresponding 95% CIs are reported. All analyses were conducted using SAS/STAT, version 9.4 (SAS Institute Inc) or R, version 4.2.3 (R Foundation for Statistical Computing). The analytic dataset was provided to the study statistician from MPOG on July 2, 2024. All primary and subgroup analyses, including evaluations by cefazolin use and surgical category, were completed by April 24, 2025.

#### Primary Analysis

For most cases, adherence was based on 3 criteria: choice of antibiotic, weight-based dose adjustment, and time of first dose. A fourth criterion was added for all cases that qualified for redosing, as the redosing guideline applied only to surgical cases with a duration of surgery greater than the primary antibiotic’s redosing interval. Cases that were nonadherent with any of the 4 metrics were considered guideline nonadherent (primary exposure).

Surgical site infection was modeled as a binary outcome at the case level, with each patient followed up for 30 days postoperatively. To examine the association of nonadherence to IDSA guidelines with SSI occurrence, hierarchical generalized linear mixed models were constructed using Poisson regression. Heterogeneity in the association between SSI and nonadherence across institutions was examined. To address nesting of cases within institutions and the potential correlation between cases within the same institution, random intercepts at the institution level were included in the modeling. Missing data (except for fraction of inspired oxygen [FIO_2_] and temperature readings, as noted later) were addressed with a multiple imputation procedure using the predicted mean matching method. Final conclusions were drawn from the pooled model results obtained from 10 imputations. Variance components were estimated using restricted maximum likelihood via the lme4 package, and final variances in multiply imputed analyses were combined using Rubin rules. As multivariable models were used to estimate the association between adherence metrics and SSI, it should be noted that other covariates included in the model functioned solely as adjustment variables and were not intended to be interpreted as independent estimators of SSI, consistent with guidance to avoid the table 2 fallacy.^20^

Because FIO_2_ and intraoperative temperature data were missing for a substantial proportion of cases (particularly monitored anesthesia care and emergency cases), we performed 2 complete case sensitivity analyses. In each analysis, we restricted the cohort to patients with complete data for the variable of interest (FIO_2_ or temperature) and reestimated the multivariable model with that variable included as an additional covariate. Another secondary analysis was conducted to evaluate and compare the association of adherence to each individual component of the guidelines. Finally, we used natural cubic splines to inspect the association between SSI incidence and timing of first dose in relation to incision.

#### Power Analysis

Based on preliminary work,^11^ we estimated that the MPOG and NSQIP sample would consist of at least 10 NSQIP centers having approximately 95 000 eligible cases for analysis. For power analysis, we assumed overall adherence to the IDSA bundle of 66% based on the prior study.^11^ Conservatively assuming that the incidence of SSIs is comparatively rare, we assumed an observed SSI incidence of between 2.0% and 2.5% in the nonadherence group. Given the aforementioned assumptions, we estimated that a sample size of 87 296 patients would achieve 80% power to detect a change of 15% relative reduction in the SSI outcome assuming an *r*^2^ of 0.2 between overall adherence and other independent variables.

## Results

### Baseline Characteristics

Of 134 413 eligible surgical cases, a total of 119 236 patients (mean [SD] age, 56.2 [15.9] years; 41.9% men and 58.1% women; 10.6% identifying as Black, 1.8% as Hispanic, 76.2% as White, 4.0% as other, and 9.3% as unknown race and ethnicity) from 37 institutions met the inclusion criteria, among whom 6786 (5.7%) had incomplete covariate data (Table 1). The majority of surgeries performed were general surgical procedures (61.1%), followed by other specialties, including gynecologic (12.7%), orthopedic (9.9%), and urologic (4.3%) surgeries. Most patients were classified as ASA class 2 (42.0%) or class 3 (48.9%), and 6.4% of cases were designated with the ASA emergency status indicator. Most demographic characteristics were well balanced between the guideline-adherent and guideline-nonadherent groups, whereas expected imbalances were observed for measures of case acuity and complexity, including ASA class, emergency status, and anesthesia duration.

**Table 1.** Baseline Demographic and Clinical Characteristics Stratified by Overall Antibiotic Use per the IDSA/SIS/SHEA Guidelines

Variable	Patients, No. (%)			SMD
	Overall cohort (N = 119 236)	Overall guideline-nonadherent cohort (n = 31 063 [26.1%])	Overall guideline-adherent cohort (n = 88 173 [73.9%])	
Age, mean (SD), y	56.2 (15.9)	56.4 (16.5)	56.1 (15.6)	0.02
Sex				
Female	69 225 (58.1)	18 167 (58.5)	51 058 (57.9)	0.01
Male	50 011 (41.9)	12 896 (41.5)	37 115 (42.1)	
Race and ethnicity				
Black	12 586 (10.6)	3158 (10.2)	9428 (10.7)	0.05
White	90 804 (76.2)	23 488 (75.6)	67 316 (76.4)	
Other^a^	4801 (4.0)	1218 (3.9)	3583 (4.1)	
Unknown	11 045 (9.3)	3199 (10.3)	7846 (8.9)	
Ethnicity				
Hispanic	2115 (1.8)	595 (1.9)	1520 (1.7)	0.05
Non-Hispanic	117 121 (98.2)	30 468 (98.1)	86 653 (98.3)	
BMI, mean (SD)	29.7 (7.5)	29.4 (8.1)	29.9 (7.3)	−0.06
ASA class				
1	5788 (4.9)	1533 (4.9)	4255 (4.8)	0.20
2	50 078 (42.0)	11 079 (35.7)	38 999 (44.2)	
3	58 257 (48.9)	16 468 (53.0)	41 789 (47.4)	
4	4956 (4.2)	1892 (6.1)	3064 (3.5)	
5	157 (0.1)	91 (0.3)	66 (0.1)	
Smoker	8613 (7.2)	2632 (8.5)	5981 (6.8)	0.06
van Walraven comorbidity index, mean (SD)	4.8 (8.5)	6.9 (9.9)	4.0 (7.8)	0.33
Diabetes	16 901 (14.2)	4873 (15.7)	12 028 (13.6)	0.06
Cerebrovascular disease	2574 (2.2)	692 (2.2)	1882 (2.1)	0.01
Year of surgery				
2014	9650 (8.1)	3592 (11.6)	6058 (6.9)	−0.11
2015	11 421 (9.6)	3113 (10.0)	8308 (9.4)	
2016	11 922 (10.0)	3035 (9.8)	8887 (10.1)	
2017	12 428 (10.4)	2943 (9.5)	9485 (10.8)	
2018	8187 (6.9)	2066 (6.7)	6121 (6.9)	
2019	8033 (6.7)	1968 (6.3)	6065 (6.9)	
2020	13 900 (11.7)	3565 (11.5)	10 335 (11.7)	
2021	26 208 (22.0)	6496 (20.9)	19 712 (22.4)	
2022	17 487 (14.7)	4285 (13.8)	13 202 (15.0)	
Anesthesia duration, mean (SD), min	218.7 (129.8)	259.5 (160.6)	204.4 (113.7)	0.40
Off-hours case (starting between 5:00 pm and 6:30 am)	6116 (5.1)	2899 (9.3)	3217 (3.7)	0.23
Medical school–affiliated center				
No	31 457 (26.4)	7782 (25.1)	23 675 (26.9)	0.04
Yes	87 779 (73.6)	23 281 (75.0)	64 498 (73.2)	
Emergency case	7652 (6.4)	4091 (13.2)	3561 (4.0)	0.34
Blood products given	3754 (3.2)	1679 (5.4)	2075 (2.4)	0.16
MAP <55 mm Hg, mean (SD), min	2.6 (7.3)	2.8 (8.0)	2.5 (7.0)	0.04
Hyperglycemia >180 mg/dL	10 280 (8.6)	3888 (12.5)	6392 (7.25)	0.19
Surgical subspecialty				
General	72 898 (61.1)	21 836 (70.3)	51 062 (57.9)	0.35
Gynecologic	15 114 (12.7)	2796 (9.0)	12 318 (14.0)	
Neurologic	4846 (4.1)	713 (2.3)	4133 (4.7)	
Oral or maxillofacial and plastic	1542 (1.3)	413 (1.3)	1129 (1.3)	
Orthopedic	11 804 (9.9)	1549 (5.0)	10 255 (11.6)	
Thoracic	3350 (2.8)	781 (2.5)	2569 (2.9)	
Urologic	5113 (4.3)	1516 (4.9)	3597 (4.1)	
Vascular	4569 (3.8)	1459 (4.7)	3110 (3.5)	

Abbreviations: ASA, American Society of Anesthesiologists; BMI, body mass index (calculated as weight in kilograms divided by height in meters squared); IDSA/SIS/SHEA, Infectious Diseases Society of America, Surgical Infection Society, and Society of Healthcare Epidemiology of America; MAP, mean arterial pressure; SMD, standardized mean difference.

SI conversion factor: To convert glucose to millimoles per liter, multiply by 0.0555.

^a^ Included American Indian or Alaska Native, Asian, and Native Hawaiian or Pacific Islander.

### Nonadherence Metrics

**Figure 1.**
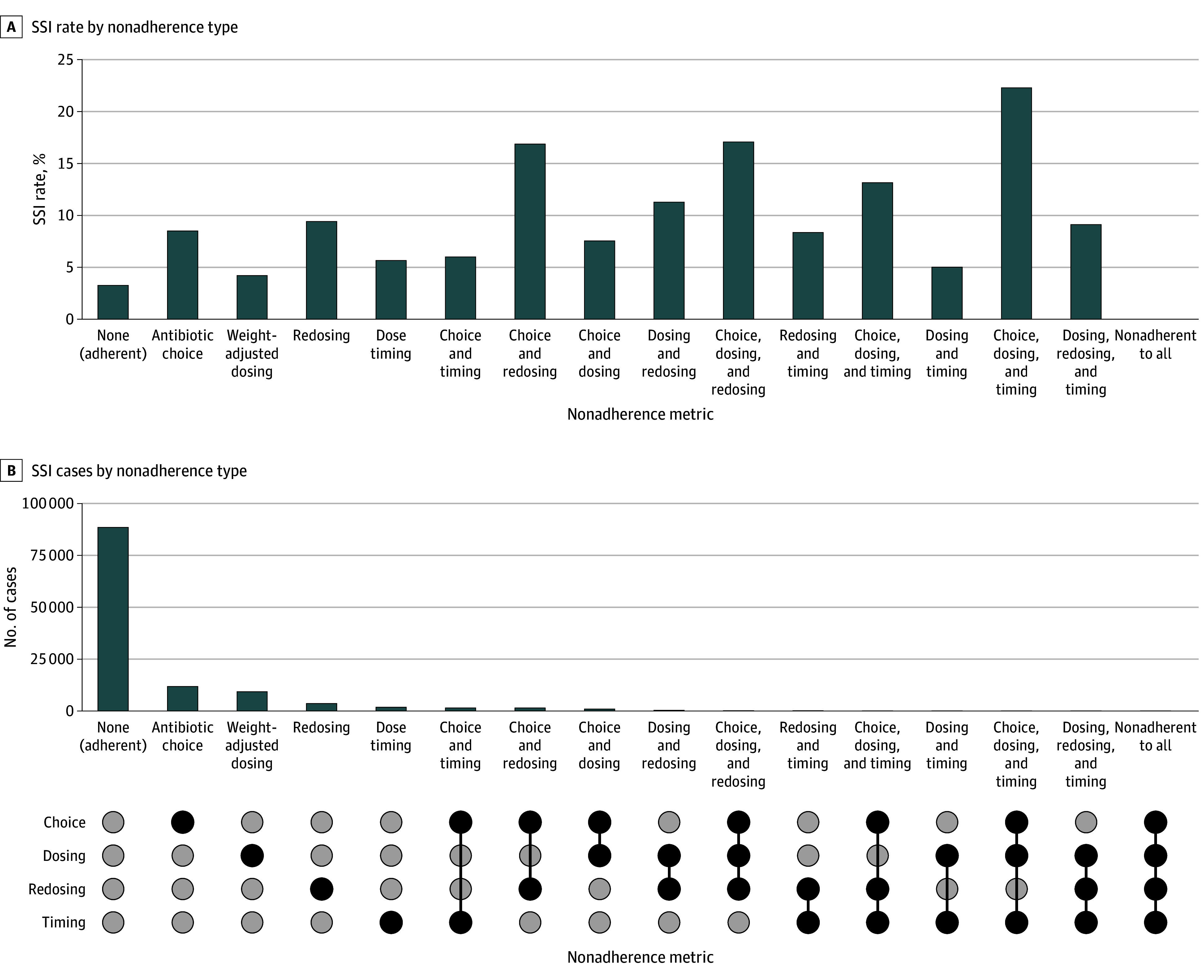
Bar Graphs of Patterns of Infectious Diseases Society of America Guideline Nonadherence and Associated Surgical Site Infection (SSI) Rates B, The UpSet-style matrix below the bar graph illustrates which antibiotic metrics were not adhered to (black circles) in each pattern.

The overall incidence of nonadherence to at least 1 antibiotic administration metric (choice, dosing, timing with respect to incision, redosing if indicated) was 31 063 (26.1%). Demographic and baseline characteristics by each antibiotic administration metric are summarized in eTables 1 to 4 in Supplement 1. The failure to adhere to individual metrics was as follows: 15 870 (13.3%) for antibiotic choice, 10 763 (9.0%) for weight-adjusted dosing, 3581 (3.0%) for timing relative to incision, and 5730 (4.8%) for redosing of antibiotic. The overall incidence of documented SSIs in the cohort was 5247 (4.4%). The incidence of SSIs with respect to nonadherence to each dosing metric is shown in Figure 1.

### Factors Associated With SSIs

**Figure 2.**
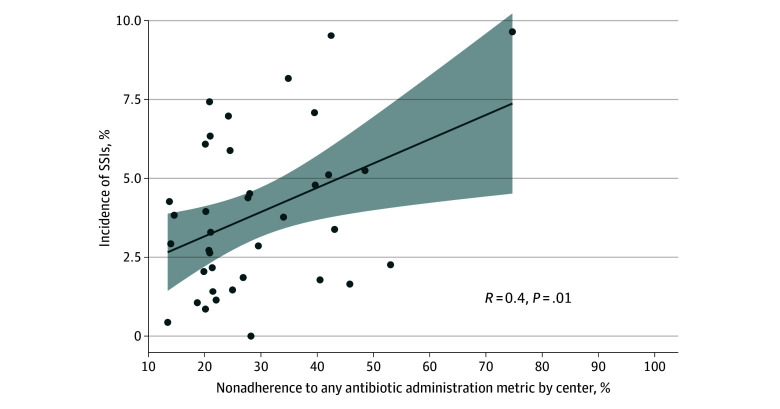
Dot Plot of the Association Between Nonadherence to Guideline-Based Antibiotic Administration and Surgical Site Infections (SSIs) Each dot indicates a center, the line indicates the best fit, and the shaded area represents the 95% CI.

On multivariable analysis (Table 2), any guideline-nonadherent antibiotic administration was significantly associated with the occurrence of SSIs (RR, 1.34 [95% CI, 1.26-1.43]; *P* < .001). Other factors also directly associated with SSIs were emergency case status (RR, 1.44 [95% CI, 1.28-1.61]; *P* < .001), off-hours case status (RR, 1.28 [95% CI, 1.13-1.45]; *P* < .001), intraoperative hyperglycemia >180 mg/dL (to convert to millimoles per liter, multiply by 0.0555) (RR, 1.17 [95% CI, 1.08-1.26]; *P* < .001), and intraoperative blood product administration (RR, 1.16 [95% CI, 1.05-1.29]; *P* = .006). An increasing ASA score (class 1 as reference) was associated with a progressively higher incidence of SSIs (class 5: RR, 3.00 [95% CI, 1.90-4.71]; class 4: RR, 2.83 [95% CI, 2.17-3.70]; class 3: RR, 2.55 [95% CI, 2.00-3.30]; class 2: RR, 1.77 [95% CI, 1.39-2.26]) (all *P* < .001). The primary model showed low levels of clustering within centers (intraclass correlation coefficient, 3%), signifying that institution-specific factors not otherwise controlled for in the primary analysis played a minimal role in the observed association. The association between overall adherence with SSI at each institution is shown in Figure 2. There was a weak, but significant trend toward a higher rate of SSIs with respect to nonadherence at the institutional level (*R* = 0.4; *P* = .01).

**Table 2.** Association of Surgical Site Infections With Demographic and Perioperative Factors and Perioperative Antibiotic Nonadherence

Variable	RR (95% CI)	*P* value
Any nonadherence	1.34 (1.26-1.43)	<.001
Age	0.99 (0.99-0.99)	<.001
Sex, male vs female	0.99 (0.93-1.05)	.62
BMI	1.01 (1.01-1.02)	<.001
Hispanic vs non-Hispanic ethnicity	0.91 (0.74-1.12)	.37
Race		
Black	0.84 (0.76-0.93)	.001
White	1 [Reference]	NA
Other^a^	0.99 (0.87-1.14)	.94
Unknown	0.94 (0.85-1.05)	.29
Surgical specialty		
Gynecologic	0.73 (0.65-0.82)	<.001
Neurologic	0.36 (0.29-0.44)	<.001
Oral or maxillofacial and plastic	0.87 (0.69-1.09)	.23
Orthopedic	0.62 (0.54-0.71)	<.001
Thoracic	0.44 (0.35-0.55)	<.001
Urologic	0.52 (0.44-0.61)	<.001
Vascular	0.66 (0.58-0.76)	<.001
General	1 [Reference]	NA
ASA class		
5	3.00 (1.90-4.71)	<.001
4	2.83 (2.17-3.70)	<.001
3	2.55 (2.00-3.30)	<.001
2	1.77 (1.39-2.26)	<.001
1	1 [Reference]	NA
van Walraven comorbidity index	1.03 (1.02-1.03)	<.001
Smoking status		
Yes	1.21 (1.07-1.37)	.002
No	1 [Reference]	NA
Diabetes	1.01 (0.94-1.10)	.74
Cerebrovascular disease	0.99 (0.83-1.20)	.92
Institution, academic vs community	1.54 (1.20-1.97)	.001
Year of surgery	1.01 (0.99-1.03)	.19
Emergency case	1.44 (1.28-1.61)	<.001
Off-hours cases (starting between 5:00 pm and 6:30 am)	1.28 (1.13-1.45)	<.001
Anesthetic duration	1.00 (1.00-1.00)	<.001
Hyperglycemia >180 mg/dL	1.17 (1.08-1.26)	<.001
Blood products given	1.16 (1.05-1.29)	.006
Mean arterial pressure <55 mm Hg	1.00 (1.00-1.00)	>.99

Abbreviations: ASA, American Society of Anesthesiologists; BMI, body mass index; NA, not applicable; RR, relative risk.

SI conversion factor: To convert glucose to millimoles per liter, multiply by 0.0555.

^a^ Included American Indian or Alaska Native, Asian, and Native Hawaiian or Pacific Islander.

### Sensitivity Analyses

We performed multiple sensitivity analyses based on our a priori plan. To assess which antibiotic metrics (choice, dosing, timing with respect to incision, redosing if indicated) were associated with SSIs, we entered each of these individually into separate multivariable models (eTable 5 in Supplement 1). Nonadherence to antibiotic choice (RR, 1.43 [95% CI, 1.33-1.53]; *P* < .001) and antibiotic redosing (RR, 1.12 [95% CI, 1.02-1.24]; *P* = .02) were significantly associated with SSIs. In this cohort, time of first dose of antibiotic was not associated with SSI. However, patients receiving antibiotics other than guideline-adherent cefazolin had a higher incidence of SSI (eFigure 2 in Supplement 1). We also performed other sensitivity analyses that included (1) only cases with complete data on temperature (n = 73 137) and (2) only cases with complete data on FIO_2_ (n = 59 834). Overall nonadherence to antibiotic administration remained significantly associated with SSIs in both models (eTables 6 and 7 in Supplement 1). Additionally, we conducted another analysis to evaluate the association of antibiotic nonadherence in surgeries that are usually associated with a relatively high risk of SSI: colorectal surgeries and hysterectomy (eFigure 3 in Supplement 1). Nonadherent antibiotic administration was associated with SSIs in colorectal surgeries but not hysterectomies.

## Discussion

This cross-sectional study of national registry data on adherence to IDSA guidelines and incidence of SSIs across multiple US health care centers found that deviations from at least 1 aspect of antibiotic guidelines were present in more than 1 of every 4 cases and were associated with a higher risk of SSIs. Higher SSI incidence in centers with higher nonadherence to antibiotic guidelines was also associated with higher SSI incidence. Among the various antibiotic administration metrics, selecting an incorrect antibiotic was the most frequent deviation and was associated with the highest likelihood of developing an SSI. Failure to redose antibiotics (when indicated) was also associated with SSIs but to a lesser degree than incorrect antibiotic type.

Despite being published more than 1 decade ago, IDSA best practice guidelines remain a source of considerable nonadherence and potential quality improvement in the perioperative episode of care.^9^ Our study highlights the critical importance of adhering to these guidelines, as deviations are associated with a higher risk of SSIs. Unlike SCIP guidelines, which primarily emphasize the timing of antibiotic administration and have near-universal adherence, IDSA guidelines are more granular, recommending appropriate antibiotic selection, dosing, and redosing for a wide array of surgeries.^21^ However, this level of detail makes adherence particularly challenging in the dynamic perioperative environment.

Notably, timing of the first antibiotic dose was not significantly associated with SSI risk in our cohort. This finding should be interpreted in the context of very high adherence to timing recommendations, with only 3% of patients receiving antibiotics outside the recommended window. In adjusted analyses, the RR for timing nonadherence was 1.13 (95% CI, 0.98-1.31), suggesting a possible, but statistically undetectable signal. Our results, combined with the higher SSI incidence observed when antibiotics other than cefazolin were used, imply that antibiotic selection may be a more influential determinant of SSI risk than timing in contemporary practice. This pattern aligns with prior studies showing no meaningful association between prophylaxis timing and SSI,^8,22,23^ although other studies have reported contrasting findings.^24,25^ One potential explanation for these mixed results is that the association between timing of the first dose and SSI risk may depend on the specific antibiotic administered due to differing pharmacokinetics. Thus, a uniform timing metric may not fully capture individualized risk across regimens.

Relevant context is provided by recent analyses from the large Swissnoso cohort.^24,26^ The Swissnoso investigators identified significant associations between timing and SSI, but their cohort exhibited much greater timing variability than ours, enhancing detectable differences. In contrast, their evaluation of weight-adjusted dosing found no increased SSI risk among patients with overweight or obesity not receiving higher doses, which aligns with our finding that weight-based dosing deviations were not major factors associated with of SSI risk. Differences in antibiotic selection (cefuroxime in Swissnoso vs primarily cefazolin in our cohort), patient mix, and adherence distributions may explain the remaining discrepancies.

Redosing has also been shown to meaningfully influence SSI risk. Bertschi et al^12^ found that failure to redose appropriately during prolonged operations increases the odds of SSI, regardless of the precise timing of antibiotic administration. Similar results were observed in 2 meta-analyses,^27,28^ which concluded that intraoperative redosing of surgical antimicrobial prophylaxis reduces SSI incidence compared with a single preoperative dose across various surgeries. However, these meta-analyses synthesized studies with substantial heterogeneity in antibiotic regimens and redosing protocols, which limited the precision of pooled estimates.

Taken together, these findings align with our results and suggest that in contemporary practice, in which adherence to SCIP timing metrics is high, improving adherence to antibiotic choice and intraoperative redosing may yield the greatest incremental benefit for SSI prevention. Several stewardship and quality-improvement interventions have successfully targeted these domains, including electronic decision support systems,^29^ pharmacist-led intraoperative antibiotic surveillance programs,^30^ and structured SSI prevention bundles emphasizing agent selection and redosing.^31^ Incorporating similar strategies into perioperative workflows may meaningfully enhance adherence and reduce SSI risk across diverse surgical settings.

Cefazolin warrants specific mention because it is the first-line prophylactic antibiotic recommended by IDSA for most clean and clean-contaminated noncardiac operations and was the predominant agent used in our cohort. As such, use of an antibiotic other than cefazolin typically reflects a deviation from guideline-concordant agent selection rather than an equivalent alternative choice for a majority of surgeries, with some notable exceptions such as positivity for methicillin-resistant *Staphylococcus aureus* or colonization with other cefazolin-resistant organisms. The higher SSI rates observed with noncefazolin regimens, therefore, may indicate instances in which nonrecommended agents were chosen despite guideline preference for cefazolin’s strong performance against common skin flora. These findings are consistent with other, similar studies.^32,33^ Although vancomycin was the second most frequent nonadherent agent and the next most commonly used antibiotic, it was associated with an SSI rate greater than the cohort average (5.6%), and many nonadherent cases reflected undercoverage when guidelines recommended multiagent prophylaxis. Thus, nonadherence was not solely driven by broad-spectrum therapy. While confounding by indication cannot be fully excluded, the similarity of demographic and comorbidity profiles across adherence groups suggests that pathogen-related factors alone may not account for the observed association.

Prior research has shown that nonadherence is especially common in emergency cases, off-hours procedures, and high-acuity surgeries requiring intraoperative interventions, such as blood transfusions.^11^ Notably, our study also found that SSIs are more prevalent in these scenarios, lending further credence to the underlying fidelity of our chosen datasets. Another known important contributor to nonadherence is the lack of a clear role among perioperative clinicians regarding who is ultimately the primary team responsible for antibiotic choice, dosing, and redosing during surgery. Existing literature has suggested that decisions related to perioperative antibiotics are often viewed as a secondary task, with no clear ownership of the decision-making process.^34,35^ Our findings underscore the urgent need to reevaluate perioperative workflows and establish clearer protocols to enhance adherence to all 4 metrics of antibiotic administration based on IDSA guidelines. Addressing these challenges through structured interventions and improved coordination, including the possibility of improved clinical decision support tools embedded within the several electronic health records currently in use may help reduce the incidence of SSIs and improve patient outcomes.

### Limitations

Our study had several limitations. First, as a retrospective observational study, it was subject to inherent limitations of the study design, including that electronic health record timestamps may have been subject to documentation variability. However, the MPOG, NSQIP, and MSQC are well-established datasets that undergo routine rigorous quality checks both at the institutional and central level.^36^ The MPOG medication timing fields undergo structured validation and auditing, and cases with missing or incomplete antibiotic timing records were excluded. Thus, the timing variable used in this analysis reflects the most reliable available data.

Second, because of the observational study design, the findings reflect adjusted associations rather than causal effects. Although we controlled for multiple clinical and procedural factors, unmeasured confounding may still influence the observed associations. Accordingly, our findings should be interpreted as identifying important patterns of association that may inform future causal investigations and quality improvement efforts.

Third, there was an absence of organism-level microbiologic data, as MPOG, NSQIP, and MSQC define SSIs based on standardized NSQIP criteria rather than culture results. Our analysis focused on adherence to IDSA prophylaxis guidelines, which are designed to prevent infections from the most common SSI pathogens, including gram-positive skin flora and, where relevant, gram-negative and anaerobic organisms through the use of alternative recommended agents. Therefore, while it remains possible that a subset of SSIs were caused by atypical or resistant pathogens requiring nonstandard prophylaxis, the balanced distribution of demographic and comorbidity characteristics across adherence groups suggests that pathogen distribution alone may not explain the observed association between guideline nonadherence and SSI risk. Additionally, although the van Walraven comorbidity index has been extensively used in similar observational studies, we could not assess nutritional status, frailty, or other potential confounders of preoperative morbidity that might influence wound healing and SSIs. Furthermore, temperature and FIO_2_ data were missing in a substantial proportion of our cohort. Since these data points were not missing at random, ie, often not recorded in cases in which general anesthesia was avoided, reflecting standard anesthetic practice, we chose not to perform imputation. However, in our sensitivity analyses using only cases with complete temperature and FIO_2_ data, the association between nonadherent antibiotic practices and SSIs remained significant. In addition, we were unable to identify patients who received extended antibiotic therapy beyond the perioperative period. This practice could have potentially reduced the observed effect size of our primary exposure by lowering the incidence of SSIs. We also were unable to capture variations in local decontamination, prepping, and draping practices across centers. However, given the low intraclass correlation coefficient of our model, these center-specific practices may not have substantially influenced our findings. Furthermore, while procedure type is an important factor associated with SSI risk, clustering at the individual procedure level would have resulted in substantial loss of power because many specific procedures had low SSI event counts. In addition, procedures would be more appropriately modeled as fixed effects rather than random effects because they represent a defined, nonrandom set of categories.

Finally, we did not include wound class as a covariate as its fidelity is controversial and has been discontinued from the NSQIP database.^37^ Despite these limitations, our findings support the importance of IDSA guideline-based antibiotic administration as a key strategy for SSI prevention.

## Conclusions

This nationwide, multicenter, cross-sectional study of patients who underwent noncardiac surgery found that IDSA guidelines (which are more comprehensive than SCIP guidelines) have suboptimal adherence despite being published more than 1 decade ago. We found that nonadherence to these guidelines is associated with a clinically significant increase in SSI risk. Implementing strategies to improve compliance, such as developing quality metric benchmarks, decision support tools within the electronic health record, and clear delineation of team responsibilities, may play a crucial role in reducing SSIs, which remain a persistent and often devastating complication for patients undergoing surgery.

## Notes

Supplement 1. **eFigure 1.** Patient Flowchart **eMethods.** Definition of 4 Metrics of Appropriate Antibiotic Administration in Accordance With IDSA Guidelines **eTable 1.** Baseline Demographic and Clinical Characteristics Stratified by Choice of Antibiotic Per the IDSA/SIS/SHEA Guidelines **eTable 2.** Baseline Demographic and Clinical Characteristics Stratified by Timing of First Dose of Antibiotic With Respect to Surgical Incision Per the IDSA/SIS/SHEA Guidelines **eTable 3.** Baseline Demographic and Clinical Characteristics Stratified by the Dose of Antibiotic Per the IDSA/SIS/SHEA Guidelines **eTable 4. **Baseline Demographic and Clinical Characteristics Stratified by the Redosing of Antibiotic Per the IDSA/SIS/SHEA Guidelines **eTable 5.** Multivariable Analysis Evaluating the Association of Surgical Site Infections With Demographic and Perioperative Factors and Each Metric of Perioperative Antibiotic Nonadherence **eFigure 2.** Association Between Time of First Antibiotic Dosing With Respect to Incision and Surgical Site Infection **eTable 6.** Multivariable Analysis Evaluating the Association of Surgical Site Infections With Demographic and Perioperative Factors and Antibiotic Nonadherence in the Cohort With Complete Temperature Data **eTable 7.** Multivariable Analysis Evaluating the Association of Surgical Site Infections With Demographic and Perioperative Factors and Antibiotic Nonadherence in the Cohort With Complete Fraction of Inspired Oxygen Data **eFigure 3.** Association of Antibiotic Nonadherence With Surgical Site Infections by Type of Surgery

Supplement 2.
Data Sharing Statement
