# Supplementary material for: Guideline Adherence of Perioperative Antibiotics and Surgical Site Infections in Noncardiac Surgery
Source: JAMA Netw Open. 2026 Feb 18;9(2):e2559349. doi: 10.1001/jamanetworkopen.2025.59349 (PMC12917684; doi:10.1001/jamanetworkopen.2025.59349)
Supplement: Supplement 1. — eFigure 1. Patient Flowchart eMethods. Definition of 4 Metrics of Appropriate Antibiotic Administration in Accordance With IDSA Guidelines eTable 1. Baseline Demographic and Clinical Characteristics Stratified by Choice of Antibiotic Per the IDSA/SIS/SHEA Guidelines eTable 2. Baseline Demographic and Clinical Characteristics Stratified by Timing of First Dose of Antibiotic With Respect to Surgical Incision Per the IDSA/SIS/SHEA Guidelines eTable 3. Baseline Demographic and Clinical Characteristics Stratified by the Dose of Antibiotic Per the IDSA/SIS/SHEA Guidelines eTable 4. Baseline Demographic and Clinical Characteristics Stratified by the Redosing of Antibiotic Per the IDSA/SIS/SHEA Guidelines eTable 5. Multivariable Analysis Evaluating the Association of Surgical Site Infections With Demographic and Perioperative Factors and Each Metric of Perioperative Antibiotic Nonadherence eFigure 2. Association Between Time of First Antibiotic Dosing With Respect to Incision and Surgical Site Infection eTable 6. Multivariable Analysis Evaluating the Association of Surgical Site Infections With Demographic and Perioperative Factors and Antibiotic Nonadherence in the Cohort With Complete Temperature Data eTable 7. Multivariable Analysis Evaluating the Association of Surgical Site Infections With Demographic and Perioperative Factors and Antibiotic Nonadherence in the Cohort With Complete Fraction of Inspired Oxygen Data eFigure 3. Association of Antibiotic Nonadherence With Surgical Site Infections by Type of Surgery [file jamanetwopen-e2559349-s001.pdf]

Supplemental Online Content

Bardia A, Lin HM, Zhao X, et al. Guideline adherence of perioperative antibiotics and surgical site infections in noncardiac surgery. *JAMA Netw Open*. 2026;9(2):e2559349
doi:10.1001/jamanetworkopen.2025.59349

eFigure 1. Patient Flowchart

eMethods. Definition of 4 Metrics of Appropriate Antibiotic Administration in Accordance With IDSA Guidelines

eTable 1. Baseline Demographic and Clinical Characteristics Stratified by Choice of Antibiotic Per the IDSA/SIS/SHEA Guidelines

eTable 2. Baseline Demographic and Clinical Characteristics Stratified by Timing of First Dose of Antibiotic With Respect to Surgical Incision Per the IDSA/SIS/SHEA Guidelines

eTable 3. Baseline Demographic and Clinical Characteristics Stratified by the Dose of Antibiotic Per the IDSA/SIS/SHEA Guidelines

eTable 4. Baseline Demographic and Clinical Characteristics Stratified by the Redosing of Antibiotic Per the IDSA/SIS/SHEA Guidelines

eTable 5. Multivariable Analysis Evaluating the Association of Surgical Site Infections With Demographic and Perioperative Factors and Each Metric of Perioperative Antibiotic Nonadherence

eFigure 2. Association Between Time of First Antibiotic Dosing With Respect to Incision and Surgical Site Infection

eTable 6. Multivariable Analysis Evaluating the Association of Surgical Site Infections With Demographic and Perioperative Factors and Antibiotic Nonadherence in the Cohort With Complete Temperature Data

eTable 7. Multivariable Analysis Evaluating the Association of Surgical Site Infections With Demographic and Perioperative Factors and Antibiotic Nonadherence in the Cohort With Complete Fraction of Inspired Oxygen Data

eFigure 3. Association of Antibiotic Nonadherence With Surgical Site Infections by Type of Surgery

This supplemental material has been provided by the authors to give readers additional information about their work.

© 2026 Bardia A et al. *JAMA Network Open*.

eFigure 1. Patient Flowchart. Adult patients who underwent surgery from 01/01/2014 to 08/31/2022 were assessed using the MPOG, NSQIP and MSQC database. After exclusions a total of 119,236 cases were included for analysis.

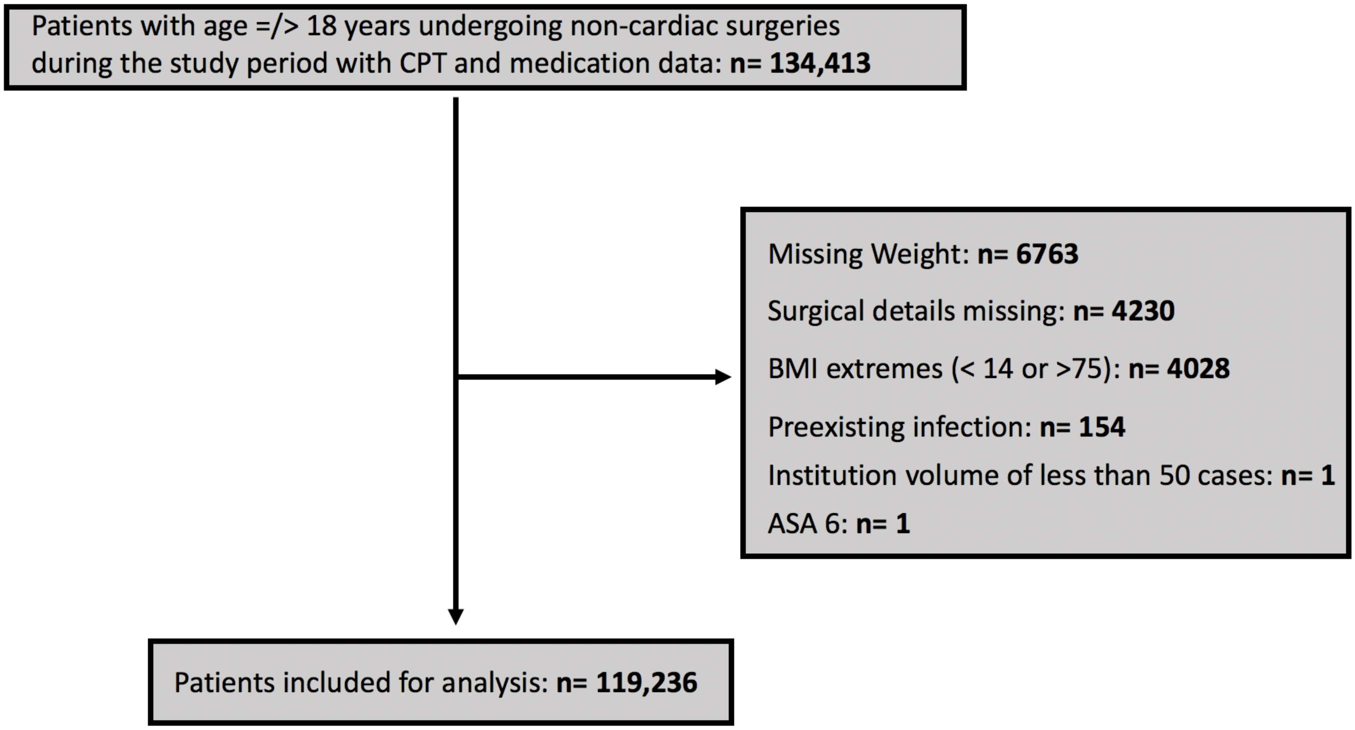

© 2026 Bardia A et al. *JAMA Network Open*.

eMethods. Definition of 4 Metrics of Appropriate Antibiotic Administration in Accordance With IDSA Guidelines

1) Appropriateness of antibiotics choice: Appropriate antibiotics according to the guidelines will be based on the procedure type using CPT codes. Furthermore, since patient/hospital-based characteristics (example drug allergies) allow for acceptable use of second line agents, we consider the antibiotic choice as appropriate if any antibiotic (first or second line) from the listed procedural category is administered.

2) Timing of antibiotic administration with respect to surgical incision: For this metric, we will use the documented time of antibiotic administration, and for infusions, we will use the start of administration as the qualifying administration time. Inappropriate timing of administration will be defined as failure to administer the antibiotic within the specific window established by the existing guidelines.

3) Timing of redosing: Instances qualifying for redosing of the antibiotic will be based on the surgical duration that is longer than the minimum interval(s) for which the guideline would indicate redosing. Successful redosing will be defined as compliance with all required additional antibiotic administrations prior to the end of surgery. Failure of any redosing will be adjudicated as failure of redosing.

4) Accuracy of dose based on weight-based adjustment: Accuracy will be based on the guidelines and considered successful if the correct antibiotic dose has been documented. For antibiotic dosing requiring mg/kg calculations (e.g., vancomycin), rounding down to no less than 75% of the estimated dose will be considered as guideline adherent.

© 2026 Bardia A et al. *JAMA Network Open*.

eTable 1. Baseline Demographic and Clinical Characteristics Stratified by Choice of Antibiotic Per the IDSA/SIS/SHEA[1] Guidelines

Variable	Overall Cohort<br>N = 119,236	Overall Guideline Non-adherent<br>N = 15,870 (13.31%)	Overall Guideline Adherent<br>N = 103,366 (86.69%)
**Age in years**, Mean (SD)	56.15 (15.87)	56.16 (16.98)	56.15 (15.70)
**Sex**			
Female	69,225 (58.06%)	8,834 (55.66%)	60,391 (58.42%)
Male	50,011 (41.94%)	7,036 (44.34%)	42,975 (41.58%)
**Hispanic Ethnicity**	2,115 (1.77%)	287 (1.81%)	1,828 (1.77%)
**Race**			
Black	12,586 (10.56%)	1,477 (9.31%)	11,109 (10.75%)
White	90,804 (76.16%)	12,204 (76.90%)	78,600 (76.04%)
Other	4,801 (4.03%)	739 (4.66%)	4,062 (3.93%)
Unknown	11,045 (9.26%)	1,450 (9.14%)	9,595 (9.28%)
White	90,805 (76.16%)	12,204 (76.90%)	78,601 (76.04%)
**BMI**, Mean (SD)	29.74 (7.49)	29.13 (7.58)	29.84 (7.47)
**ASA Status**			
ASA Class 1	5,788 (4.85%)	924 (5.82%)	4,864 (4.71%)
ASA Class 2	50,078 (42.00%)	5,322 (33.53%)	44,756 (43.30%)
ASA Class 3	58,257 (48.86%)	8,495 (53.53%)	49,762 (48.14%)
ASA Class 4	4,956 (4.16%)	1,066 (6.72%)	3,890 (3.76%)
ASA Class 5	157 (0.13%)	63 (0.40%)	94 (0.09%)
**Smoker**	8,613 (7.22%)	1,517 (9.56%)	7,096 (6.86%)
**Van Walraven Comorbidity Index,** Mean (SD)	4.75 (8.51)	7.75 (10.29)	4.29 (8.10)

© 2026 Bardia A et al. *JAMA Network Open*.

Variable	Overall Cohort N = 119,236	Overall Guideline Non-adherent N = 15,870 (13.31%)	Overall Guideline Adherent N = 103,366 (86.69%)
**Diabetes**	16,901 (14.17%)	2,625 (16.54%)	14,276 (13.81%)
**Cerebrovascular Disease**	2,574 (2.16%)	273 (1.72%)	2,301 (2.23%)
**Year of Surgery**			
2014	9,650 (8.09%)	1,212 (7.64%)	8,438 (8.16%)
2015	11,421 (9.58%)	1,327 (8.36%)	10,094 (9.77%)
2016	11,922 (10.00%)	1,281 (8.07%)	10,641 (10.29%)
2017	12,428 (10.42%)	1,217 (7.67%)	11,211 (10.85%)
2018	8,187 (6.87%)	1,039 (6.55%)	7,148 (6.92%)
2019	8,033 (6.74%)	1,228 (7.74%)	6,805 (6.58%)
2020	13,900 (11.66%)	2,210 (13.93%)	11,690 (11.31%)
2021	26,208 (21.98%)	3,865 (24.35%)	22,343 (21.62%)
2022	17,487 (14.67%)	2,491 (15.70%)	14,996 (14.51%)
**Anesthesia Duration** (minutes), Mean (SD)	218.74 (129.82)	255.29 (166.95)	213.13 (122.16)
**Off Hour Case** (Starting between 5PM and 6:30AM)	6,116 (5.13%)	2,140 (13.48%)	3,976 (3.85%)
**Medical School Affiliated Center**			
No	31,457 (26.38%)	4,514 (28.44%)	26,943 (26.07%)
Yes	87,779 (73.62%)	11,356 (71.56%)	76,423 (73.93%)
**Emergency Case**	7,652 (6.42%)	3,125 (19.69%)	4,527 (4.38%)
**Blood Products Given**	3,754 (3.15%)	869 (5.48%)	2,885 (2.79%)

© 2026 Bardia A et al. *JAMA Network Open*.

Variable	Overall Cohort N = 119,236	Overall Guideline Non-adherent N = 15,870 (13.31%)	Overall Guideline Adherent N = 103,366 (86.69%)
**MAP < 55 (minutes),** Mean (SD)	2.55 (7.25)	2.73 (7.39)	2.52 (7.22)
**Hyperglycemia** (>180 mg/dl)	10,280 (8.62%)	2,220 (13.99%)	8,060 (7.80%)
**Subspecialty**			
General Surgery	72,898 (61.14%)	12,865 (81.06%)	60,033 (58.08%)
Gynecology	15,114 (12.68%)	724 (4.56%)	14,390 (13.92%)
Neurosurgery	4,846 (4.06%)	64 (0.40%)	4,782 (4.63%)
OMFS/Plastics	1,542 (1.29%)	144 (0.91%)	1,398 (1.35%)
Orthopedics	11,804 (9.90%)	341 (2.15%)	11,463 (11.09%)
Thoracic Surgery	3,350 (2.81%)	407 (2.56%)	2,943 (2.85%)
Urology	5,113 (4.29%)	802 (5.05%)	4,311 (4.17%)
Vascular Surgery	4,569 (3.83%)	523 (3.30%)	4,046 (3.92%)

1: IDSA/SIS/SHEA: Infectious Diseases Society of America, SIS: Surgical Infection Society, SHEA: Society of Healthcare Epidemiology of America;

© 2026 Bardia A et al. *JAMA Network Open*.

eTable 2. Baseline Demographic and Clinical Characteristics Stratified by Timing of First Dose of Antibiotic With Respect to Surgical Incision Per the IDSA/SIS/SHEA[1] Guidelines

Variable	Overall Cohort N = 119,236	Overall Guideline Non-adherent N = 3,581 (3.00%)	Overall Guideline Adherent N = 115,655 (97.00%)
**Age in years**, Mean (SD)	56.15 (15.87)	54.39 (18.10)	56.20 (15.80)
**Sex**			
Female	69,225 (58.06%)	1,995 (55.71%)	67,230 (58.13%)
Male	50,011 (41.94%)	1,586 (44.29%)	48,425 (41.87%)
**Hispanic Ethnicity**	2,115 (1.77%)	102 (2.85%)	2,013 (1.74%)
**Race**			
Black	12,586 (10.56%)	434 (12.12%)	12,152 (10.51%)
White	90,804 (76.16%)	2,729 (76.21%)	88,075 (76.15%)
Other	4,801 (4.03%)	123 (3.43%)	4,678 (4.04%)
Unknown	11,045 (9.26%)	295 (8.24%)	10,750 (9.29%)
**BMI**, Mean (SD)	29.74 (7.49)	29.89 (7.71)	29.74 (7.48)
**ASA Status**			
ASA Class 1	5,788 (4.85%)	221 (6.17%)	5,567 (4.81%)
ASA Class 2	50,078 (42.00%)	1,176 (32.84%)	48,902 (42.28%)
ASA Class 3	58,257 (48.86%)	1,825 (50.96%)	56,432 (48.79%)
ASA Class 4	4,956 (4.16%)	328 (9.16%)	4,628 (4.00%)
ASA Class 5	157 (0.13%)	31 (0.87%)	126 (0.11%)
**Smoker**	8,613 (7.22%)	464 (12.96%)	8,149 (7.05%)
**Van Walraven Comorbidity Index,** Mean(SD)	4.75 (8.51)	6.72 (9.95)	4.69 (8.45)
**Diabetes**	16,901 (14.17%)	606 (16.92%)	16,295 (14.09%)

© 2026 Bardia A et al. *JAMA Network Open*.

Variable	Overall Cohort N = 119,236	Overall Guideline Non-adherent N = 3,581 (3.00%)	Overall Guideline Adherent N = 115,655 (97.00%)
**Cerebrovascular Disease**	2,574 (2.16%)	115 (3.21%)	2,459 (2.13%)
**Year of Surgery**			
2014	9,650 (8.09%)	39 (1.09%)	9,611 (8.31%)
2015	11,421 (9.58%)	74 (2.07%)	11,347 (9.81%)
2016	11,922 (10.00%)	45 (1.26%)	11,877 (10.27%)
2017	12,428 (10.42%)	47 (1.31%)	12,381 (10.71%)
2018	8,187 (6.87%)	71 (1.98%)	8,116 (7.02%)
2019	8,033 (6.74%)	249 (6.95%)	7,784 (6.73%)
2020	13,900 (11.66%)	724 (20.22%)	13,176 (11.39%)
2021	26,208 (21.98%)	1,409 (39.35%)	24,799 (21.44%)
2022	17,487 (14.67%)	923 (25.77%)	16,564 (14.32%)
**Anesthesia Duration** (minutes), Mean (SD)	218.74(129.82)	201.74(144.65)	219.27(129.30)
**Off Hour Case** (Starting between 5PM and 6:30AM)	6,116 (5.13%)	811 (22.65%)	5,305 (4.59%)
**Medical School Affiliated Center**			
No	31,457 (26.38%)	1,892 (52.83%)	29,565 (25.56%)
Yes	87,779 (73.62%)	1,689 (47.17%)	86,090 (74.44%)
**Emergency Case**	7,652 (6.42%)	1,081 (30.19%)	6,571 (5.68%)
**Blood Products Given**	3,754 (3.15%)	189 (5.28%)	3,565 (3.08%)

© 2026 Bardia A et al. *JAMA Network Open*.

Variable	Overall Cohort N = 119,236	Overall Guideline Non-adherent N = 3,581 (3.00%)	Overall Guideline Adherent N = 115,655 (97.00%)
**MAP < 55 (minutes),** Mean (SD)	2.55 (7.25)	2.00 (6.15)	2.56(7.28)
**Hyperglycemia** (>180 mg/dl)	10,280 (8.62%)	389 (10.86%)	9,891 (8.55%)
**Subspecialty**			
General Surgery	72,898 (61.14%)	2,765 (77.21%)	70,133 (60.64%)
Gynecology	15,114 (12.68%)	149 (4.16%)	14,965 (12.94%)
Neurosurgery	4,846 (4.06%)	125 (3.49%)	4,721 (4.08%)
OMFS/Plastics	1,542 (1.29%)	15 (0.42%)	1,527 (1.32%)
Orthopedics	11,804 (9.90%)	252 (7.04%)	11,552 (9.99%)
Thoracic Surgery	3,350 (2.81%)	38 (1.06%)	3,312 (2.86%)
Urology	5,113 (4.29%)	74 (2.07%)	5,039 (4.36%)
Vascular Surgery	4,569 (3.83%)	163 (4.55%)	4,406 (3.81%)

1: IDSA/SIS/SHEA: Infectious Diseases Society of America, SIS: Surgical Infection Society, SHEA: Society of Healthcare Epidemiology of America;

© 2026 Bardia A et al. *JAMA Network Open*.

eTable 3. Baseline Demographic and Clinical Characteristics Stratified by the Dose of Antibiotic Per the IDSA/SIS/SHEA[1] Guidelines

Variable	Overall Cohort N = 119,236	Overall Guideline Non-adherent N = 10,763 (9.03%)	Overall Guideline Adherent N = 108,474 (90.97%)
**Age in years**, Mean (SD)	56.15 (15.87)	56.57 (16.26)	56.11 (15.83)
**Sex**			
Female	69,225 (58.06%)	6,957 (64.64%)	62,268 (57.40%)
Male	50,011 (41.94%)	3,806 (35.36%)	46,205 (42.60%)
**Hispanic Ethnicity**	2,115 (1.77%)	209 (1.94%)	1,906 (1.76%)
**Race**			
Black	12,586 (10.56%)	1,108 (10.29%)	11,478 (10.58%)
White	90,804 (76.16%)	7,933 (73.71%)	82,871 (76.40%)
Other	4,801 (4.03%)	335 (3.11%)	4,466 (4.12%)
Unknown	11,045 (9.26%)	1,387 (12.89%)	9,658 (8.90%)
**BMI**, Mean (SD)	29.74 (7.49)	29.27 (9.04)	29.79 (7.31)
**ASA Status**			
ASA Class 1	5,788 (4.85%)	559 (5.19%)	5,229 (4.82%)
ASA Class 2	50,078 (42.00%)	4,278 (39.75%)	45,800 (42.22%)
ASA Class 3	58,257 (48.86%)	5,341 (49.62%)	52,916 (48.78%)
ASA Class 4	4,956 (4.16%)	569 (5.29%)	4,387 (4.04%)
ASA Class 5	157 (0.13%)	16 (0.15%)	141 (0.13%)
**Smoker**	8,613.00 (7.22%)	735 (6.83%)	7,878 (7.26%)
**Van Walraven Comorbidity Index,** Mean(SD)	4.75 (8.51)	5.07 (8.69)	4.72 (8.49)
**Diabetes**	16,901 (14.17%)	1,421 (13.20%)	15,480 (14.27%)
**Cerebrovascular Disease**	2,574 (2.16%)	275 (2.56%)	2,299 (2.12%)

© 2026 Bardia A et al. *JAMA Network Open*.

Variable	Overall Cohort<br>N = 119,236	Overall Guideline Non-adherent<br>N = 10,763 (9.03%)	Overall Guideline Adherent<br>N = 108,474 (90.97%)
**Year of Surgery**			
2014	9,650 (8.09%)	2,325 (21.60%)	7,325 (6.75%)
2015	11,421 (9.58%)	1,495 (13.89%)	9,926 (9.15%)
2016	11,922 (10.00%)	1,426 (13.25%)	10,496 (9.68%)
2017	12,428 (10.42%)	1,362 (12.65%)	11,066 (10.20%)
2018	8,187 (6.87%)	584 (5.43%)	7,603 (7.01%)
2019	8,033 (6.74%)	288 (2.68%)	7,745 (7.14%)
2020	13,900 (11.66%)	739 (6.87%)	13,161 (12.13%)
2021	26,208 (21.98%)	1,569 (14.58%)	24,639 (22.71%)
2022	17,487 (14.67%)	975 (9.06%)	16,512 (15.22%)
**Anesthesia Duration** (minutes), Mean (SD)	218.74(129.82)	228.72(136.40)	217.75 (129.10)
**Off Hour Case** (Starting between 5PM and 6:30AM)	6,116 (5.13%)	542 (5.04%)	5,574 (5.14%)
**Medical School Affiliated Center**			
No	31,457 (26.38%)	2,381 (22.12%)	29,076 (26.80%)
Yes	87,779 (73.62%)	8,382 (77.88%)	79,397 (73.20%)
**Emergency Case**	7,652 (6.42%)	652 (6.06%)	7,000 (6.45%)
**Blood Products Given**	3,754 (3.15%)	458 (4.26%)	3,296 (3.04%)
**MAP < 55 (minutes),** Mean (SD)	2.55 (7.25)	2.87 (8.77)	2.52 (7.09)
**Hyperglycemia** (>180 mg/dl)	10,280 (8.62%)	856 (7.95%)	9,424 (8.69%)

© 2026 Bardia A et al. *JAMA Network Open*.

Variable	Overall Cohort N = 119,236	Overall Guideline Non-adherent N = 10,763 (9.03%)	Overall Guideline Adherent N = 108,474 (90.97%)
**Subspecialty**			
General Surgery	72,898 (61.14%)	6,390 (59.37%)	66,508 (61.31%)
Gynecology	15,114 (12.68%)	1,432 (13.30%)	13,682 (12.61%)
Neurosurgery	4,846 (4.06%)	440 (4.09%)	4,406 (4.06%)
OMFS/Plastics	1,542 (1.29%)	201 (1.87%)	1,341 (1.24%)
Orthopedics	11,804 (9.90%)	869 (8.07%)	10,935 (10.08%)
Thoracic Surgery	3,350 (2.81%)	316 (2.94%)	3,034 (2.80%)
Urology	5,113 (4.29%)	518 (4.81%)	4,595 (4.24%)
Vascular Surgery	4,569 (3.83%)	597 (5.55%)	3,972 (3.66%)

1: IDSA/SIS/SHEA: Infectious Diseases Society of America, SIS: Surgical Infection Society, SHEA: Society of Healthcare Epidemiology of America;

© 2026 Bardia A et al. *JAMA Network Open*.

eTable 4. Baseline Demographic and Clinical Characteristics Stratified by the Redosing of Antibiotic Per the IDSA/SIS/SHEA[1] Guidelines

Variable	Overall Cohort N = 119,236	Overall Guideline Non-adherent N = 5,730 (4.81%)	Overall Guideline Adherent N = 113,506 (95.19%)
**Age in years**, Mean (SD)	56.15(15.87)	58.69(14.73)	56.02(15.92)
**Sex**			
Female	69,225(58.06%)	3,019 (52.69%)	66,206 (58.33%)
Male	50,011 (41.94%)	2,711 (47.31%)	47,300 (41.67%)
**Hispanic Ethnicity**	2,115 (1.77%)	83 (1.45%)	2,032 (1.79%)
**Race**			
Black	12,586 (10.56%)	593 (10.35%)	11,993 (10.57%)
White	90,804 (76.16%)	4,371 (76.28%)	86,433 (76.15%)
Other	4,801 (4.03%)	223 (3.89%)	4,578 (4.03%)
Unknown	11,045 (9.26%)	543 (9.48%)	10,502 (9.25%)
**BMI**, Mean (SD)	29.74(7.49)	29.61(7.26)	29.75(7.50)
**ASA Status**			
ASA Class 1	5,788 (4.85%)	77 (1.34%)	5,711 (5.03%)
ASA Class 2	50,078 (42.00%)	1,763 (30.77%)	48,315 (42.57%)
ASA Class 3	58,257 (48.86%)	3,586 (62.58%)	54,671 (48.17%)
ASA Class 4	4,956 (4.16%)	292 (5.10%)	4,664 (4.11%)
ASA Class 5	157 (0.13%)	12.00 (0.21%)	145 (0.13%)
**Smoker**	8,613 (7.22%)	497 (8.67%)	8,116 (7.15%)
**Van Walraven Comorbidity Index,** Mean(SD)	4.75 (8.51)	9.68 (10.87)	4.50 (8.29)
**Diabetes**	16,901 (14.17%)	1,073 (18.73%)	15,828 (13.94%)

© 2026 Bardia A et al. *JAMA Network Open*.

Variable	Overall Cohort N = 119,236	Overall Guideline Non-adherent N = 5,730 (4.81%)	Overall Guideline Adherent N = 113,506 (95.19%)
**Cerebrovascular Disease**	2,574 (2.16%)	138 (2.41%)	2,436 (2.15%)
**Year of Surgery**			
2014	9,650 (8.09%)	550 (9.60%)	9,100 (8.02%)
2015	11,421 (9.58%)	608 (10.61%)	10,813 (9.53%)
2016	11,922 (10.00%)	691 (12.06%)	11,231 (9.89%)
2017	12,428 (10.42%)	690 (12.04%)	11,738 (10.34%)
2018	8,187 (6.87%)	666 (11.62%)	7,521 (6.63%)
2019	8,033 (6.74%)	571 (9.97%)	7,462 (6.57%)
2020	13,900 (11.66%)	554 (9.67%)	13,346 (11.76%)
2021	26,208 (21.98%)	749 (13.07%)	25,459 (22.43%)
2022	17,487 (14.67%)	651 (11.36%)	16,836 (14.83%)
**Anesthesia Duration** (minutes), Mean (SD)	218.74 (129.82)	393.53 (152.19)	209.92 (122.12)
**Off Hour Case** (Starting between 5PM and 6:30AM)	6,116 (5.13%)	169 (2.95%)	5,947 (5.24%)
**Medical School Affiliated Center**			
No	31,457 (26.38%)	498 (8.69%)	30,959 (27.27%)
Yes	87,779 (73.62%)	5,232 (91.31%)	82,547 (72.73%)
**Emergency Case**	7,652 (6.42%)	352 (6.14%)	7,300 (6.43%)
**Blood Products Given**	3,754 (3.15%)	555 (9.69%)	3,199 (2.82%)
**MAP < 55 (minutes),** Mean (SD)	2.55 (7.25)	3.14 (8.58)	2.52 (7.17)
**Hyperglycemia**	10,280 (8.62%)	1,267 (22.11%)	9,013 (7.94%)

© 2026 Bardia A et al. *JAMA Network Open*.

Variable	Overall Cohort N = 119,236	Overall Guideline Non-adherent N = 5,730 (4.81%)	Overall Guideline Adherent N = 113,506 (95.19%)
(>180 mg/dl)			
**Subspecialty**			
General Surgery	72,898 (61.14%)	3,708 (64.71%)	69,190 (60.96%)
Gynecology	15,114 (12.68%)	652 (11.38%)	14,462 (12.74%)
Neurosurgery	4,846 (4.06%)	132 (2.30%)	4,714 (4.15%)
OMFS/Plastics	1,542 (1.29%)	92 (1.61%)	1,450 (1.28%)
Orthopedics	11,804 (9.90%)	156 (2.72%)	11,648 (10.26%)
Thoracic Surgery	3,350 (2.81%)	112 (1.95%)	3,238 (2.85%)
Urology	5,113 (4.29%)	428 (7.47%)	4,685 (4.13%)
Vascular Surgery	4,549 (3.83%)	450 (7.85%)	4,119 (3.63%)

1: IDSA/SIS/SHEA: Infectious Diseases Society of America, SIS: Surgical Infection Society, SHEA: Society of Healthcare Epidemiology of America;

© 2026 Bardia A et al. *JAMA Network Open*.

eTable 5. Multivariable Analysis Evaluating the Association of Surgical Site Infections With Demographic and Perioperative Factors and Each Metric of Perioperative Antibiotic Nonadherence

Variable	Relative Risk (95% CI)	p-Value
Non-adherent choice	**1.43 (1.33, 1.53)**	**<0.001**
Non-adherent dosing	1.10 (0.99-1.22)	0.068
Non-adherent timing	1.13 (0.98-1.31)	0.087
Non-adherent re-dosing	**1.12 (1.02-1.24)**	**0.020**
Age (in years)	**0.99 (0.99, 0.99)**	**<0.001**
Sex, Male vs Female	0.98 (0.93, 1.05)	0.604
BMI (kg/m^2)	**1.01 (1.01, 1.02)**	**<0.001**
Hispanic vs. Non-Hispanic	0.91 (0.74, 1.11)	0.348
Race		
Black	**0.84 (0.76, 0.94)**	**0.001**
Others	0.99 (0.87, 1.14)	0.938
Unknown	0.94 (0.84, 1.05)	0.258
White	Ref	
Surgical Specialty		
Gynecology	**0.74 (0.67, 0.83)**	**<0.001**
Neurosurgery	**0.38 (0.30, 0.46)**	**<0.001**
OMFS/Plastics	0.90 (0.71, 1.13)	0.348
Orthopedics	**0.63 (0.55, 0.72)**	**<0.001**
Thoracic Surgery	**0.44 (0.35, 0.56)**	**<0.001**
Urology	**0.52 (0.44, 0.61)**	**<0.001**
Vascular Surgery	**0.67 (0.59, 0.77)**	**<0.001**
General Surgery	Ref	
ASA class		
5	**3.01 (1.91, 4.74)**	**<0.001**
4	**2.86 (2.19, 3.73)**	**<0.001**
3	**2.57 (2.02, 3.29)**	**<0.001**
2	**1.79 (1.41, 2.28)**	**<0.001**
1	Ref	
Van Walraven Comorbidity Index	**1.03 (1.02, 1.03)**	**<0.001**
Smoking Status (ref. No)	**1.20 (1.06, 1.36)**	**0.003**
Diabetes	1.01 (0.94, 1.10)	0.766
Cerebrovascular Disease	1.00 (0.84, 1.20)	0.977
Institution, Academic vs. Community	**1.54 (1.20, 1.97)**	**0.001**
Year of Surgery	1.01 (0.99, 1.03)	0.335
Emergency case	**1.40 (1.25, 1.58)**	**<0.001**
Off-hours Cases (Starting between 5PM and 6:30AM)	**1.26 (1.11, 1.43)**	**<0.001**
Anesthetic Duration (minutes)	1.00 (1.00, 1.00)	**<0.001**

© 2026 Bardia A et al. *JAMA Network Open*.

Hyperglycemia (>180 mg/dl)	**1.16 (1.07, 1.26)**	**<0.001**
Blood products given	**1.17 (1.05, 1.30)**	**0.005**
MAP <55	1.00 (1.00, 1.00)	0.924

© 2026 Bardia A et al. *JAMA Network Open*.

eFigure 2. Association Between Time of First Antibiotic Dosing With Respect to Incision and Surgical Site Infection. There was a lower incidence of SSI in the patients who received cefazolin (red line) as compared to those who did not (blue line). There was no discernable trend of change in incidence of SSI with respect to time of first dose of antibiotic administration. Shaded areas represent 95% confidence interval.

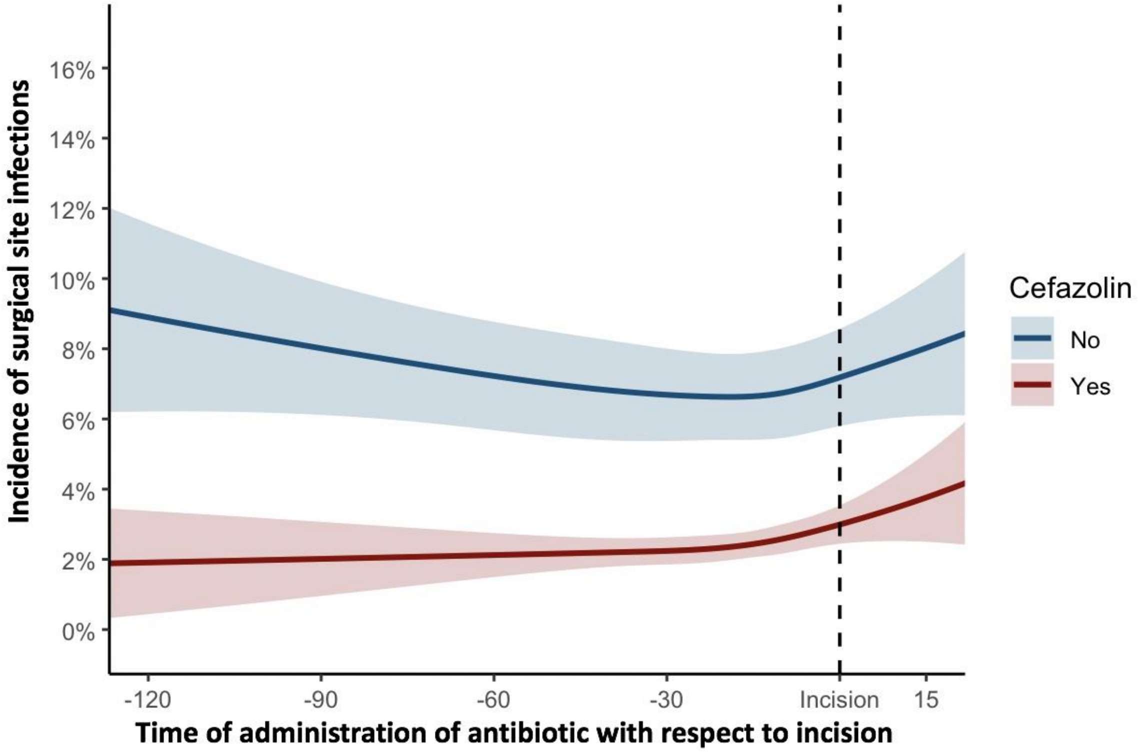

© 2026 Bardia A et al. *JAMA Network Open*.

eTable 6. Multivariable Analysis Evaluating the Association of Surgical Site Infections With Demographic and Perioperative Factors and Antibiotic Nonadherence in the Cohort With Complete Temperature Data (n = 72,891)

Variable	Relative Risk (95% CI)	p-Value
Overall antibiotic non-adherence	1.34 (1.24, 1.44)	**<0.001**
Age (in years)	1.00 (1.00, 1.00)	0.066
Sex, Male vs Female	0.95 (0.88, 1.02)	0.131
BMI (kg/m^2)	1.01 (1.01, 1.02)	**<0.001**
Hispanic vs. Non-Hispanic	0.99 (0.75, 1.30)	0.938
Race		
Black	0.87 (0.77, 0.98)	**0.021**
Others	1.03 (0.88, 1.21)	0.708
Unknown	0.94 (0.81, 1.08)	0.367
White	Ref	
Surgical Specialty		
Gynecology	0.73 (0.63, 0.84)	**<0.001**
Neurosurgery	0.40 (0.30, 0.53)	**<0.001**
OMFS/Plastics	0.82 (0.60, 1.11)	0.204
Orthopedics	0.80 (0.68, 0.96)	**0.014**
Thoracic Surgery	0.39 (0.29, 0.52)	**<0.001**
Urology	0.49 (0.40, 0.60)	**<0.001**
Vascular Surgery	0.71 (0.60, 0.84)	**<0.001**
General Surgery	Ref	
ASA class		
5	2.40 (1.31, 4.40)	**0.004**
4	2.54 (1.83, 3.55)	**<0.001**
3	2.40 (1.77, 3.26)	**<0.001**
2	1.72 (1.27, 2.33)	**<0.001**
1	Ref	
Van Walraven Comorbidity Index	1.03 (1.02, 1.03)	**<0.001**
Smoking Status (ref. No)	1.20 (1.06, 1.37)	**0.005**
Diabetes	0.92 (0.84, 1.01)	0.092
Cerebrovascular Disease	1.00 (0.81, 1.24)	0.991
Institution, Academic vs. Community	2.06 (1.54, 2.76)	**<0.001**
Year of Surgery	1.02 (0.99, 1.04)	0.153
Emergency case	1.30 (1.14, 1.48)	**<0.001**
Off-hours Cases (Starting between 5PM and 6:30AM)	1.16 (1.00, 1.36)	0.053
Anesthetic Duration (minutes)	1.00 (1.00, 1.00)	**<0.001**
Hyperglycemia (>180 mg/dl)	1.51 (1.34, 1.71)	**<0.001**
Blood products given	1.15 (1.01, 1.30)	**0.032**
MAP <55	1.00 (1.00, 1.01)	0.263

© 2026 Bardia A et al. *JAMA Network Open*.

Temperature over the median[1] (36.1 C)	1.24 (1.18, 1.31)	**<0.001**

1. Median temperature (IQR) of the cohort: 36.1(35.80, 36.60) degree C.

© 2026 Bardia A et al. *JAMA Network Open*.

eTable 7. Multivariable Analysis Evaluating the Association of Surgical Site Infections With Demographic and Perioperative Factors and Antibiotic Nonadherence in the Cohort With Complete Fraction of Inspired Oxygen Data (n = 59644)

Variable	Relative Risk (95% CI)	p-Value
Overall antibiotic non-adherence	1.38 (1.27, 1.50)	**<0.001**
Age (in years)	0.99 (0.99, 1.00)	**<0.001**
Sex, Male vs Female	1.01 (0.93, 1.09)	0.882
BMI (kg/m^2)	1.01 (1.01, 1.02)	**<0.001**
Hispanic vs. Non-Hispanic	0.79 (0.59, 1.04)	0.097
Race		
Black	0.86 (0.76, 0.98)	**0.022**
Others	0.98 (0.78, 1.24)	0.867
Unknown	0.92 (0.79, 1.07)	0.298
White	Ref	
Surgical Specialty		
Gynecology	0.64 (0.55, 0.75)	**<0.001**
Neurosurgery	0.35 (0.28, 0.44)	**<0.001**
OMFS/Plastics	0.88 (0.65, 1.20)	0.430
Orthopedics	0.60 (0.52, 0.70)	**<0.001**
Thoracic Surgery	0.49 (0.37, 0.65)	**<0.001**
Urology	0.52 (0.43, 0.63)	**<0.001**
Vascular Surgery	0.69 (0.59, 0.82)	**<0.001**
General Surgery	Ref	
ASA class		
5	2.65 (1.46, 4.82)	**0.001**
4	2.16 (1.48, 3.15)	**<0.001**
3	2.12 (1.49, 3.01)	**<0.001**
2	1.52 (1.07, 2.16)	**0.018**
1	Ref	
Van Walraven Comorbidity Index	1.03 (1.02, 1.03)	**<0.001**
Smoking Status (ref. No)	1.10 (0.94, 1.30)	0.247
Diabetes	0.98 (0.88, 1.08)	0.667
Cerebrovascular Disease	0.95 (0.76, 1.18)	0.622
Institution, Academic vs. Community	2.57 (1.73, 3.82)	**<0.001**
Year of Surgery	0.97 (0.95, 1.00)	**0.017**
Emergency case	1.20 (1.03, 1.41)	**0.023**
Off-hours Cases (Starting between 5PM and 6:30AM)	1.19 (0.98, 1.43)	0.074
Anesthetic Duration (minutes)	1.00 (1.00, 1.00)	**<0.001**
Hyperglycemia (>180 mg/dl)	1.41 (1.24, 1.60)	**<0.001**
Blood products given	1.19 (1.04, 1.37)	**0.012**
MAP <55	1.00 (0.99, 1.00)	0.197

© 2026 Bardia A et al. *JAMA Network Open*.

Fraction of inspired oxygen (FiO2)	1.00 (1.00, 1.00)	0.340

© 2026 Bardia A et al. *JAMA Network Open*.

eFigure 3. Association of Antibiotic Nonadherence With Surgical Site Infections by Type of Surgery

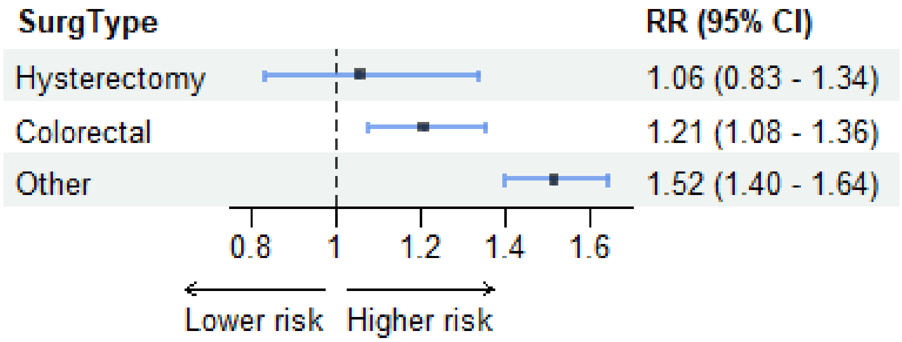

© 2026 Bardia A et al. *JAMA Network Open*.
