# Supplementary material for: Guideline Adherence of Perioperative Antibiotics and Surgical Site Infections in Noncardiac Surgery
Source: JAMA Netw Open. 2026 Feb 18;9(2):e2559349. doi: 10.1001/jamanetworkopen.2025.59349 (PMC12917684; doi:10.1001/jamanetworkopen.2025.59349)
Supplement: Supplement 2. — Data Sharing Statement [file jamanetwopen-e2559349-s002.pdf]

Data Sharing Statement

Data
Data available: No

Additional Information
Explanation for why data not available: The consortium doesnot allow for data sharing.
